# Lagged and simultaneous effects of exposure to violence at home on child-to-parent violence: gender differences

**DOI:** 10.3389/fpsyt.2024.1441871

**Published:** 2024-09-02

**Authors:** M. Carmen Cano-Lozano, María J. Navas-Martínez, Lourdes Contreras

**Affiliations:** Department of Psychology, University of Jaén, Jaén, Spain

**Keywords:** child-to-parent violence, exposure to violence at home, direct victimization, vicarious victimization, adolescents, gender

## Abstract

**Introduction:**

Numerous studies have found that exposure to violence at home is a risk factor for child-to-parent violence. However, most of the available studies do not delimit a time frame for exposure to violence. This aspect is fundamental to differentiating lagged effects (compensation) from simultaneous effects (reciprocal). The purpose of this study is to clarify the relationship between lagged (before the age of 10) and simultaneous (last year) exposure to violence at home (direct victimization: parent-to-child violence and vicarious victimization: exposure to violence between parents) and child-to-parent violence, the possible differential reactive or instrumental motivation of these relationships and whether they differ based on the gender of children and parents.

**Method:**

The sample comprised 1,734 Spanish adolescents who lived with both parents (57.3% girls), aged between 13 and 17 years. The instruments used were the Child-to-Parent Violence Questionnaire and the Violence Exposure Scale.

**Results:**

Positive and significant relationships were found between child-to-parent violence and exposure to violence at home both during childhood and during the last year; however, the relationships were stronger in the latter. The most important predictors were direct parental victimization during the last year. Boys exerted more reactive violence toward the father concerning exposure to violence by the father toward the mother during the last year. In the case of girls, violence toward both father and mother is more reactive to most victimization experiences.

**Conclusions:**

The findings highlight the need to intervene in family contexts of violence to prevent child-to-parent violence.

## 1 Introduction

The violence of children towards parents, known as child-to-parent violence (hereafter CPV), is a form of family violence that has garnered significant concern due to its spectacular increase in recent years and its serious consequences. Research on the topic has grown substantially at the international level, generating abundant information on various risk factors at the individual, family, and social levels (1). Among these factors, exposure to violence is one of the most empirically supported (2). In fact, exposure to violence is a variable that has traditionally been associated with the development of violent behaviors, especially during adolescence (e.g., 3, 4).

In regard to exposure to violence in different contexts, empirical studies with judicial populations indicate that CPV young offenders present higher levels of exposure to violence at home compared to other young offenders (5–7). An interesting result is that other offenders presented higher levels of exposure to violence in the community than CPV offenders (5). Thus, what differentiates these groups of offenders is the context in which exposure to violence occurs, confirming that exposure to family violence may play a crucial role in violent behaviors from children towards parents. The relationship between CPV and exposure to violence at home has also been consistently observed in studies with community populations (8–15). The results of numerous studies on CPV are consistent with this approach, finding that a significant proportion of adolescents who abuse their parents have been exposed to situations of family violence. Specifically, a recent study with a sample of 3,142 adolescents from a community population found that more than half of the adolescents who exercise CPV have experienced some type of violence within the family context (54.9%) (16). Similar results were found in another study with a judicial sample, which reported that 54% of adolescent perpetrators of child-to-mother violence had witnessed family violence, and 25% had suffered direct victimization by their parents (17). A meta-analytic review examining the literature on the relationship between child-to-parent violence and parent-to-child violence, noted that the probability of developing CPV for children victimized by parents increased by 71% compared to non-victimized children (2).

Exposure to violence in the family context includes both direct and vicarious victimization. Direct victimization occurs when children are victims of parental violence, and vicarious victimization occurs when children witness violence between parents. Some research has analyzed both types of victimization in CPV separately. Studies with community population have found a relationship between CPV and both direct family victimization (10, 13, 18–24) and vicarious family victimization (10, 13, 18, 21, 23, 25). Likewise, both direct and vicarious victimization are significant predictors of this type of violence (9, 10, 13, 26). Parent-to-child violence explains 16.8% of CPV, compared to 13.7% explained by exposure to violence between parents (2). More specifically, it has been found that mother-to-child violence (15.8%) and, although to a lesser extent, exposure to violence by the mother toward the father, jointly explain 17% of child-to-mother violence. On the other hand, father-to-child violence (17.5%) and, although to a lesser extent, exposure to violence by the mother to the father, jointly explain almost 19% of child-to-father violence (10). These data suggest reciprocity in CPV and that the aggressive behaviors of young people toward their parents may represent responses to previous aggressions by the parents. Thus, direct victimization has a greater predictive capacity for CPV than vicarious victimization (8, 9, 13). In studies involving judicial populations, it is found that, compared to other offenders, CPV offenders exhibit more direct victimization at home (5, 20) and that both CPV offenders and CPV non-offenders show higher levels of direct and indirect victimization at home than young non-CPV (27).

In theoretical terms, the social learning theory (28, 29) provides an explanatory framework for CPV. Children from violent homes may acquire aggressive patterns of responses through observation, learning, and reinforcement of aggressive adult models. Consequently, they may resort to violence as a solution for coping with interpersonal conflicts. Two complementary models derived from social learning theory have been proposed to explain the relationship between child and parental violence (30). In model 1, parent-to-child violence (Time 1) predicts CPV (Time 2), and subsequently, CPV inhibits parent-to-child violence. There are unidirectional and lagged effects (compensation). In model 2 there are simultaneous or close in time effects between parent-to-child and CPV (bidirectional violence) (2). Despite receiving broad support, the experimental designs used in the studies do not sufficiently validate the two models derived from this theory. Most research comprises cross-sectional studies that fail to delimit a time frame for exposure to violence. Therefore, it is imperative to clearly specify the time frame to which exposure to violence refers to differentiate the distant effects from the simultaneous or immediate effects.

A first approach to this issue was undertaken in the study by Brezina (30). This study conducted analyses using data from the first and second waves of data collection (approximately one and a half years apart) from a national survey of 15-year-old adolescent boys. The data collected included information on parental and child physical aggression. Although the results point to a reciprocal relationship between parental and child aggression, the survey data used were over 30 years old, referred to single-age adolescent boys, and the measures were based on a single indicator (1 item referring to physical aggression). Therefore, the authors themselves approached the study rather as an initial hypothesis test.

On the other hand, a more recent retrospective study analyzes the effects of exposure to violence at home during childhood (before the age of 10 years) on CPV that occurs during adolescence (between the ages of 13 and 18 years old). It is concluded that both direct victimization and vicarious victimization independently contribute to CPV, which could indicate that CPV may be a distant effect of exposure to violence at home during childhood (18).

The occurrence of exposure to violence in the familiar context does not explain, on its own, how adolescents come to behave violently toward their parents. It is necessary to understand the underlying motivations of the violence that adolescents exert towards their parents in this type of situation. Theses aspects may be of great importance to understand the possible explanatory mechanisms involved (compensation vs. reciprocal). Regarding the motivation for violence, a distinction has been made between reactive violence which refers to the use of violence in response to a previous aggression or threat of aggression, and instrumental violence which involves the use of violence to get what one wants (31). In the context of CPV, several studies have evaluated the reasons why these types of violent behaviors are committed, finding the presence of both types of reasons (10, 32). More specifically, regarding exposure to violence at home, a study with community adolescents revealed that exposure to violence at home is related to CPV motivated by reactive and instrumental reasons (11). More specifically, direct victimization at home is related to CPV only for reactive reasons in specialist aggressors (showing only CPV) whereas in generalist aggressors (showing other types of violence in addition to CPV) for both reactive and instrumental reasons (33).

These results regarding direct victimization at home are consistent with findings from a study conducted with a judicial sample (20). In this study, although direct victimization at home was related to both reactive and instrumental CPV through different socio-cognitive variables, the weight of reactive reasons was greater than the weight of instrumental reasons. Concerning the time frame of exposure to violence, it is expected that direct victimization during the last year (simultaneous or close in time) is more related to reactive CPV, which would support the potential reciprocal or bidirectional effects of this violence. However, there are no studies that analyze this aspect.

On the other hand, it is necessary to pay attention to gender differences. Research on CPV has pointed out differences according to the gender of children and parents. Regarding differences according to the gender of children, studies conducted with community samples developed in Germany and the United States have found that girls exhibit higher levels of verbal abuse compared to boys (19) and, more specifically, towards mothers (8). Studies carried out in Spain indicate that, in general, girls exert more psychological violence than boys (10, 34, 35) and, specifically, towards mothers (15). Studies involving judicial samples have indicated that girls exhibit more physical violence and more control and domain behaviors towards mothers compared to boys (20). Regarding the reasons for CPV, gender differences have also been identified, with girls showing higher levels of reactive reasons than boys (16, 20, 33). When considering differences based on the gender of parents, there is considerable consensus in the scientific literature on the role of the mother as the main victim (8, 15, 35).

Differences have also been detected between what boys and girls report in relation to exposure to violence at home. Armstrong et al. (36), in a judicial study using secondary data, observed that girls among the group of CPV offenders reported more direct victimization. In another study with a judicial sample, Cano-Lozano et al. (20) found that girls reported more direct and vicarious victimization at home than boys. However, studies with community samples do not reveal conclusive data. In the study of Calvete et al. (15), boys and girls reported similar levels of exposure to violence at home. Izaguirre & Calvete (26) found that while daughters were more likely to experience psychological victimization, sons were more likely to experience physical victimization. In another study, the girls reported higher levels of vicarious victimization (19). More specifically, Cano-Lozano et al. (10) found that girls reported more direct victimization by mothers and more vicarious victimization by fathers toward mothers compared to boys.

In short, it is necessary to analyze in a differentiated form the immediate and distant effects of exposure to violence at home to determine whether both influence and have the same explanatory weight in the CPV. It is also important to know the reactive or instrumental motivations underlying CPV, based on the time in which exposure to violence occurs. Likewise, in line with previous research that identifies gender differences in both CPV and exposure to violence at home, studies that address gender differences in these relationships are needed.

Specifically, this research intends to clarify the relationship between lagged (before 10 years) and simultaneous (last year) exposure to violence at home (direct victimization: parent-to-child violence and vicarious victimization: exposure to violence between parents) and child-to-parent violence, the possible differential reactive or instrumental motivation of these relationships and if they differ depending on the gender of the children and parents.

Specifically, the following objectives and hypotheses are proposed:

-Examine the relationships between CPV (toward the father and the mother) and lagged and simultaneous direct and vicarious family victimization (father and mother) in the case of both boys and girls. A positive and significant relationship is expected between CPV and direct and vicarious victimization, being the strongest relationship with direct victimization (H1) (13, 18, 19, 21).

-Identify the types of family victimization that best predict CPV towards the father and mother, both in the case of boys and girls. It is expected that direct and vicarious victimization are significant predictors of CPV (9, 13, 14, 26), with direct victimization being the predictor with the greatest explanatory capacity (H2) (9, 13). Lagged (18) and simultaneous (30) effects of exposure to violence at home on CPV are also expected, although it is unclear which effect would have greater explanatory capacity due to the absence of studies.

-Analyze what type of reasons for CPV (reactive, instrumental, or both) intervene in the relationship between the different types of family victimization (lagged and simultaneous) and CPV, and whether these results are different for boys and girls. Direct victimization, especially in the last year, is expected to be related to CPV more through reactive than instrumental reasons (H3) (20).

## 2 Method

### 2.1 Participants

The initial sample consisted of 2,124 participants, of whom those living with both the father and the mother were selected for this study. The final sample consisted of 1,734 adolescents (57.3% girls) of Spanish nationality (97.7%) aged between 13 and 17 years (*M*_age_ = 14.9, *SD* = 1.3) recruited from 25 educational centers located in four Spanish regions in the south (33.6%), center (61.1%) and north (5.3%) of Spain. Of the sample, 99.2% were biological children and most of the participants’ parents were married (96.5%). The socioeconomic levels were as follows: 10.5% high, 58% medium- sufficient, and 3.9 low-sufficient.

### 2.2 Instruments

Child-to-parent violence and reasons of child-to-parent violence: *Child-to-Parent Violence Questionnaire, adolescent version* (*CPV-Q*, 37). This *Spanish* instrument assesses the frequency of a series of violent behaviors (psychological, physical, economic, and control/domain) exercised during the last year toward the father (α = .68) and toward the mother (α = .72) through 14 parallel items that are answered using a 5-point Likert scale (0 = *never* to 4 = *very often, six times or more*). This instrument also includes adolescents’ reactive (α = .81) and instrumental (α = .83) reasons for carrying out CPV behaviors through 8 parallel items that are answered using a 4-point Likert scale (0 = *never* to 3 = *always*). The global CPV score is obtained by adding the scores of the 14 items of the father (CPV father) and 14 items of the mother (CPV mother).

Exposure to violence at home: *Violence Exposure Scale, adapted version* (*VES*, 32). It is a Spanish 21-item questionnaire that assesses both direct and vicarious exposure to violence in the contexts of home, school, street, and TV. For this study, only the exposure to violence at home subscale was used. An adaptation was made for this study including the parental figures involved (father-to-child violence, mother-to-child violence, father-to-mother violence and mother-to-father violence) and two different time frames (T1: during childhood, before the age of 10 years, and T2: during the last year). Specifically, for both temporal moments, direct violence (psychological, physical, and verbal) by parents were assessed through 3 parallel items (direct victimization of father and mother) and observed violence (psychological, physical, and verbal) between parents through 3 parallel items (vicarious victimization of father to mother and mother to father) answered using a 5-point Likert scale (0 = *never* to 4 = *every day*). An example of an item assessing direct victimization would be “How often has your father/mother hit or physically harmed you? while for vicarious victimization, an example would be “How often have you seen how your father/mother hit or physically harmed your mother/father? The global score for direct victimization and vicarious victimization was obtained by adding the scores of the 3 items, respectively. In this studio, internal consistency ranges from acceptable to good. Specific, Cronbach’s alpha for direct victimization was α = .87 during the past year and α = .88 during childhood. For vicarious victimization, it was α = .71 during the past year and α = .78 during childhood.

### 2.3 Procedure

First, a favorable report was obtained from the Ethics Committee of the University of Jaén to carry out the research (reference: OCT.19/1.PRY). Subsequently, authorizations were obtained from the Public Administration in the field of Education and from the educational centers involved who were invited to participate and received detailed information about the research. When selecting the educational centers, the type of school was taken into account (public vs. private) as well as the school year of the adolescents (between 13 and 17 years old). The secondary schools that expressed their interest to participate in the study provided and collected informed consent on paper from both parents and children. Participation was voluntary and consisted of completing a battery of questionnaires for approximately one hour. These questionnaires on paper were administered by researchers specifically trained in this protocol, in person and in groups, within the educational classrooms themselves. Each student was assigned an anonymous identification code. No incentives were provided for participation in the study.

### 2.4 Data analysis

A significance level of 0.05 was established for all tests. Initially, the relationships between the study variables (CPV, reasons for CPV, and exposure to violence at home) were examined for both boys and girls through correlational analyses (see **Table 1**).

**Table 1:** Correlations between study variables.

		1	2	3	4	5	6	7	8	9	10	11	12	13	14
1	CPV-F	–	.85^***^	.51^***^	.47^***^	.48^***^	.45^***^	.32^***^	.31^***^	.28^***^	.27^***^	.25^***^	.27^***^	.20^***^	.27^***^
2	CPV-M	.85^***^	–	.45^***^	.54^***^	.44^***^	.55^***^	.33^***^	.39^***^	.33^***^	.34^***^	.27^***^	.27^***^	.25^***^	.28^***^
3	RR-F	.49^***^	.45^***^	–	.80^***^	.31^***^	.31^***^	.50^***^	.42^***^	.38^***^	.31^***^	.38^***^	.38^***^	.27^***^	.31^***^
4	RR-M	.43^***^	.50^***^	.84^***^	–	.28^***^	.35^***^	.39^***^	.55^***^	.30^***^	.43^***^	.32^***^	.39^***^	.25^***^	.34^***^
5	IR-F	.47^***^	.50^***^	.30^***^	.27^***^	–	.83^***^	.18^***^	.21^***^	.15^***^	.16^***^	.10^**^	.15^***^	.05	.08^*^
6	IR-M	.46^***^	.56^***^	.28^***^	.31^***^	.87^***^	–	.20^***^	.29^***^	.17^***^	.22^***^	.13^***^	.17^***^	.06	.12^***^
7	T2DV-F	.40^***^	.41^***^	.43^***^	.36^***^	.25^***^	.27^***^	–	.70^***^	.68^***^	.44^***^	.48^***^	.37^***^	.36^***^	.27^***^
8	T2DV-M	.32^***^	.38^***^	.33^***^	.40^***^	.20^***^	.23^***^	.74^***^	–	.45^***^	.65^***^	.37^***^	.46^***^	.22^***^	.32^***^
9	T1DV-F	.37^***^	.37^***^	.30^***^	.27^***^	.22^***^	.25^***^	.68^***^	.56^***^	–	.68^***^	.38^***^	.27^***^	.50^***^	.36^***^
10	T1DV-M	.29^***^	.32^***^	.24^***^	.32^***^	.16^***^	.21^***^	.53^***^	.70^***^	.79^***^	–	.28^***^	.38^***^	.29^***^	.44^***^
11	T2VV-FM	.26^***^	.30^***^	.35^***^	.35^***^	.16^***^	.21^***^	.48^***^	.34^***^	.37^***^	.29^***^	–	.73^***^	.73^***^	.57^***^
12	T2VV-MF	.27^***^	.31^***^	.31^***^	.36^***^	.19^***^	.22^***^	.39^***^	.45^***^	.32^***^	.38^***^	.78^***^	–	.51^***^	.72^***^
13	T1VV-FM	.26^***^	.30^***^	.27^***^	.25^***^	.17^***^	.21^***^	.41^***^	.26^***^	.45^***^	.32^***^	.78^***^	.63^***^	–	.70^***^
14	T1VV-MF	.22^***^	.28^***^	.24^***^	.26^***^	.22^***^	.24^***^	.31^***^	.33^***^	.36^***^	.40^***^	.63^***^	.78^***^	.78^***^	–

CPV, Child-to-Parent violence; F, Father; M, Mother; RR, Reactive Reasons; IR, Instrumental Reasons; T1, Time frame referring to childhood; T2, Time frame referring to the last year; DV, Direct Victimization; VV, Vicarious Victimization; FM, Father-to-Mother; MF, Mother-to-Father. Results for the sample of boys (n = 740) are shown below the diagonal and those for the sample of girls (n = 994) are shown above the diagonal.

*p < 0.05, **p < 0.01, ***p < 0.001.

Subsequently, a multiple linear regression analysis was also performed independently in boys and girls for each dependent variable (violence toward the father and violence toward the mother). Each independent variable was included in individual blocks, and entered hierarchically according to their degree of theoretical relevance (38). Specifically, in the first and second blocks, direct victimization of fathers and mothers during the last year, respectively, was added. In the third and fourth blocks, this type of victimization that occurred during childhood was added. In the fifth and sixth blocks, vicarious victimization from fathers to mothers and from mothers to fathers during the last year was added, and in the seventh and eighth blocks, the same type of victimization that occurred during childhood was added. In addition, given the nature of the predictors, the “stepwise” method for independent variable selection was used to control potential multicollinearity issues by ensuring the elimination of redundant predictors in the equation. The change in the *R* ^2^ statistic was also needed to check the value added by each predictor retained in the equation. This approach allows us to discriminate the temporal moment in which victimization occurs and the figures involved that best predict CPV, both in the case of boys and girls (see **Table 2**).

**Table 2:** Multiple linear regressions of child-to-parent violence against fathers and mothers as a function of children’s gender.

	Boys										Girls									
	Model Summary					Coefficient					Model Summary					Coefficient				
	Model	*R^2^*	Δ*R* ^2^	Δ*F*	*df*	Predictors	*B*	*SE*	β	*t*	Model	*R^2^*	Δ*R* ^2^	Δ*F*	*df*	Predictors	*B*	*SE*	β	*t*
Child-to-Father Violence	1	.167	.166	137.93^***^	689						1	.097	.096	98.94^***^	926					
	2	.190	.187	19.44^***^	688						2	.112	.110	15.68^***^	925					
	3	.201	.197	9.40^**^	687						3	.121	.118	10.17^**^	924					
						(Intercept)	3.44	0.17		20.60^***^	4	.126	.122	5.20^*^	923					
						T2DV-F	0.44	0.10	.22	4.38^***^	5	.134	.130	8.85^**^	922					
						T1DV-F	0.39	0.09	.20	4.38^***^	6	.141	.135	6.47^*^	921					
						T2VV-FM	0.52	0.17	.12	3.07^**^	7	.148	.142	8.07^**^	920					
																(Intercept)	3.96	0.18		22.50^***^
																T2DV-F	0.32	0.13	.15	2.44^*^
																T1VV-MF	0.59	0.21	.13	2.84^**^
Child-to-Mother Violence	1	.178	.177	149.60^***^	689						1	.096	.095	97.90^***^	926					
	2	.188	.185	8.00^**^	688						2	.155	.153	64.57^***^	925					
	3	.202	.199	12.33^***^	687						3	.175	.173	23.23^***^	924					
	4	.230	.226	24.94^***^	686						4	.186	.183	12.20^**^	923					
	5	.239	.233	7.72^**^	685						5	.191	.186	5.12^*^	922					
						(Intercept)	3.70	0.17		19.92^***^	6	.194	.189	3.92^*^	921					
						T2DV-F	0.33	0.13	.15	2.51^*^						(Intercept)	4.36	0.18		24.19^***^
						T2DV-M	0.31	0.12	.13	2.59^**^						T2DV-M	0.71	0.09	.33	7.60^***^
						T1DV-F	0.25	0.10	.12	2.47^*^						T1DV-F	0.28	0.09	.14	3.06^**^
						T1VV-FM	0.66	0.24	.15	2.78^**^						T1VV-MF	0.41	0.21	.09	1.98^*^

T1, Time frame referring to childhood; T2, Time frame referring to the last year; DV, Direct Victimization; VV, Vicarious Victimization; F, Father; M, Mother; FM, Father-to-Mother; MF, Mother-to-Father.

*p < 0.05, **p < 0.01, ***p < 0.001.

**Figure 1:**
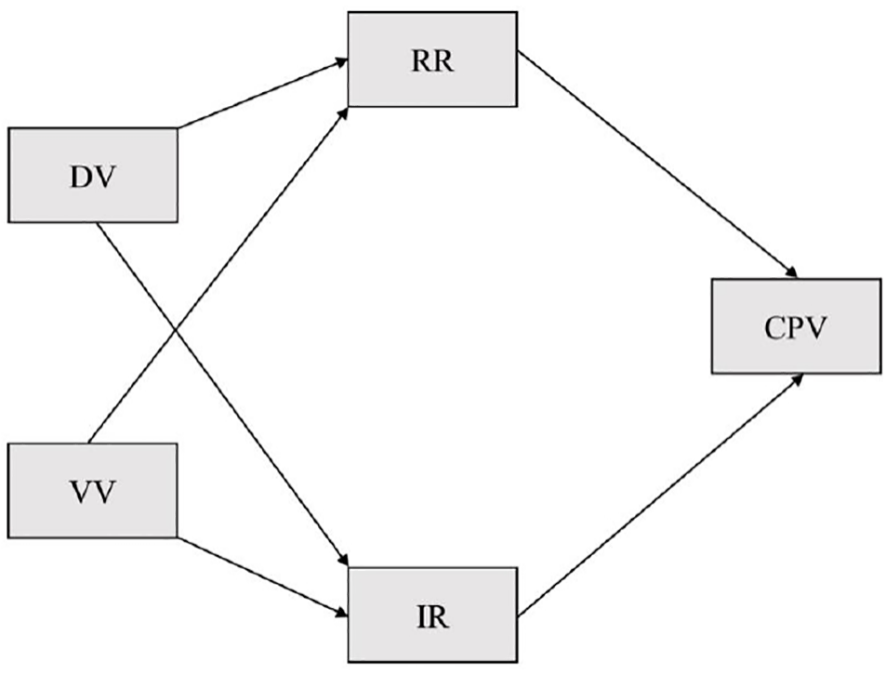
Proposed mediation theoretical model. DV, Direct Victimization; VV, Vicarious Victimization; RR, Reactive Reasons; IR, Instrumental Reason; CPV, Child-to-Parent Violence.

Prior to conducting the analysis, the regression assumptions were assessed. The significance of the bivariate correlation coefficients allowed us to accept the assumption of linearity between the dependent and independent variables. The absence of multicollinearity between the predictors retained in the equation (VIF < 2.5) and the independence of the residuals generated (Durbin-Watson between 1.5 and 2.5) was also verified. However, there are issues regarding the normality and homoscedasticity of the residuals. Specifically, scatter plots of standardized residuals reveal that the standardized residuals are distributed heteroscedastically along the predicted standardized scores. Additionally, the significance of the Kolmogorov-Smirnov test with Lilliefors correction (*p* < 0.05) shows that the standardized residuals do not follow a multivariate normal distribution. Violation of these two assumptions should not be problematic in cross-sectional studies and with sample size exceeding 30 (39). Furthermore, in this community-based study, the absence of normality and homoscedasticity is explained by the asymmetric dispersion between the values of the variables analyzed given that by definition in this type of population, a large proportion of adolescents do not show or experience violence, which is reflected in asymmetric distributions that are common in community studies on CPV (e.g., 9, 40). Finally, the indirect relationships of the model proposed in **Figure 1** were analyzed.

We proposed to analyze a) what type of CPV reasons: reactive, instrumental, or both, are involved in the relationship between the types of victimization analyzed: direct and vicarious victimization during childhood (T1) and during the last year (T2) and CPV and b) whether they differ according to the type of victimization and parent involved in the victimization, and c) whether these results are different for boys and girls (see **Table 3**). Before conducting the analysis, the assumptions of correlations between (1) predictor and dependent, (2) predictor and mediator, and (3) mediator and dependent variables were checked (see **Table 1**). To assess the significance of indirect effects, we used the Sobel test and the 95% corrected confidence intervals generated from bootstrapping estimates with a total of 10,000 resamples (significant indirect effects if the confidence intervals did not contain the value 0).

**Table 3:** Results of the indirect effects of the reasons for child-to-parent violence.

	Boys								Girls										
	Model	Path			95% CI ^1^				Model	Path						95% CI ^1^			
			β	*SE*	LL	UL	*z*	*p*						β	*SE*	LL	UL	*z*	*p*
Child-to-Father Violence	1	T2DV-F → RR → CPV	.14	0.02	0.10	0.18	7.71	< .001	1	T2DV-F → RR → CPV				.18	0.02	0.14	0.22	9.84	< .001
		T2DV-F → IR → CPV	.09	0.02	0.06	0.13	5.92	< .001		T2DV-F → IR → CPV				.06	0.01	0.04	0.09	4.83	< .001
		(C1) RR – IR	.05	0.03	-0.01	0.10				(C1) RR – IR				.12	0.03	0.06	0.17		
	2	T1DV-F → RR → CPV	.10	0.02	0.07	0.14	6.60	< .001	2	T1VV-MF → RR → CPV				.12	0.02	0.08	0.15	8.21	< .001
		T1DV-F → IR → CPV	.08	0.02	0.05	0.11	5.05	< .001		T1VV-MF → IR → CPV				.03	0.01	0.01	0.06	2.38	.017
		(C1) RR – IR	.02	0.03	-0.03	0.07				(C1) RR – IR				.09	0.02	0.04	0.13		
	3	T2VV-FM → RR → CPV	.12	0.02	0.08	0.16	7.36	< .001											
		T2VV-FM → IR → CPV	.06	0.02	0.02	0.10	4.00	< .001											
		(C1) RR – IR	.06	0.03	0.002	0.11													
Child-to-Mother Violence	1	T2DV-F → RR → CPV	.10	0.02	0.08	0.14	7.31	< .001	1	T2DV-M → RR → CPV				.20	0.02	0.16	0.24	10.48	< .001
		T2DV-F → IR → CPV	.11	0.02	0.07	0.17	6.47	< .001		T2DV-M → IR → CPV				.12	0.02	0.08	0.15	7.87	< .001
		(C1) RR – IR	-.01	0.03	-0.06	0.05				(C1) RR – IR				.08	0.03	0.02	0.14		
	2	T2DV-M → RR → CPV	.12	0.02	0.09	0.16	7.76	< .001	2	T1DV-F → RR → CPV				.11	0.02	0.08	0.15	7.99	< .001
		T2DV-M → IR → CPV	.10	0.02	0.06	0.15	5.88	< .001		T1DV-F → IR → CPV				.07	0.02	0.04	0.12	5.05	< .001
		(C1) RR – IR	.02	0.03	-0.04	0.08				(C1) RR – IR				.04	0.02	-0.00	0.09		
	3	T1DV-F → RR → CPV	.09	0.02	0.06	0.12	6.20	< .001	3	T1VV-MF → RR → CPV				.13	0.02	0.09	0.17	8.58	< .001
		T1DV-F → IR → CPV	.10	0.02	0.06	0.14	6.38	< .001		T1VV-MF → IR → CPV				.05	0.02	0.02	0.08	3.66	< .001
		(C1) RR – IR	-.02	0.02	-0.06	0.03				(C1) RR – IR				.08	0.02	0.04	0.12		
	4	T1VV-FM → RR → CPV	.08	0.02	0.05	0.12	5.96	< .001											
		T1VV-FM → IR → CPV	.09	0.03	0.04	0.16	5.34	< .001											
		(C1) RR – IR	-.01	0.03	-0.08	0.05													

T1, Time frame referring to childhood; T2, Time frame referring to the last year; DV, Direct Victimization; VV, Vicarious Victimization; F, Father; M, Mother; FM, Father to Mother; MF, Mother to Father; RR, Reactive Reasons; RI, Instrumental Reasons; CPV, Child-to-Parent Violence. The reasons for each model (CPV toward father and CPV toward mother) refer to the reasons for violence toward both figures separately. (C1) = Difference between the indirect effect of RR and the indirect effect of IR.

^1^ Bootstrapping results with confidence intervals for the lower (LLCI) and upper limits (ULCI).

## 3 Results

CPV is positively and significantly related to direct and vicarious victimization both during the last year and during childhood, for both boys and girls (see **Table 1**). In general, in both samples, CPV presents higher correlation coefficients with direct victimization (*r* = .27 to *r* = .40) than with vicarious victimization (*r* = .20 to *r* = .30), and in turn, also higher with victimization during the last year than with victimization during childhood. Moreover, regarding direct victimization, the results differentiated by gender suggest a certain degree of reciprocity between perpetrated violence and experienced violence, and in turn, a certain correspondence according to the gender of children and parents. Specifically, in boys, CPV toward fathers correlates slightly stronger with victimization by father (*r* = .40) than with victimization by mother (*r* = .32), while in girls, CPV toward mother correlates slightly stronger with victimization by mother (*r* = .39) than with victimization by father (*r* = .33).

Regarding CPV reasons, both reactive and instrumental reasons correlate positively and significantly with both direct and vicarious victimization, both during the last year and during childhood, for both boys and girls, with one exception (see **Table 1**). Specifically, in the girls, instrumental reasons do not correlate significantly with vicarious victimization (father to mother) during childhood. The results also show that both types of victimization have higher correlation coefficients with reactive reasons (*r* = .24 to *r* = .55) than with instrumental reasons (*r* = .08 to *r* = .29). Positive and significant correlations are also found between reactive and instrumental reasons and CPV toward the father and mother in both boys and girls.

Regression analyses (see **Table 2**) show which types of victimization best predict CPV toward fathers and mothers in boys and girls independently. Both direct victimization and vicarious victimization are significant predictors of CPV in both samples, although there are differences in the type of victimization, in the parents involved, and in the temporal context, depending on gender.

In the case of violence toward the father, the proportion of variance explained by the retained variables in the models differ between boys and girls, being slightly higher in the sample of boys (*R* ^2^ = .201) than in the sample of girls (*R* ^2^ = .148). Specifically, direct victimization by the father is a common predictor of both samples, as well as the best predictor, while the predictors referring to vicarious victimization are specific to each sample. In the case of boys, the predictor variable is exposure to violence from the father to the mother (during the last year) while in the case of girls, it is exposure to violence from the mother to the father (during childhood).

Regarding violence toward the mother, the proportion of variance explained by the variables finally retained in the models is also slightly higher in the sample of boys (*R^2^* = .239) than in the sample of girls (*R^2^* = .194). Specifically, direct victimization by both parents (by the mother during the last year and by the father during childhood) are common predictors of child-to-mother violence in both samples. However, only in the boys’ sample does direct victimization by the father (during the last year) also predict violence toward the mother. Regarding vicarious victimization, similar to the results for violence toward the father, violence toward the mother is explained in boys by exposure to violence from the father toward the mother and in girls by exposure to violence from the mother toward the father, although in this case, both occurred during childhood.

Overall, in the case of boys, the best predictor of violence toward the father and toward the mother is the victimization by father during the last year. In the case of girls, the best predictor of violence toward the father is the victimization by father and the best predictor of violence toward the mother is the victimization by mother, both referring to the last year.

Finally, the results regarding the indirect effects of reactive and instrumental reasons for CPV on the relationship between different types of victimization and CPV demonstrate that both types of reasons are involved in this relationship. However, these results once again vary by gender.

In boys, violence towards the father responds to both reactive and instrumental reasons to victimization by the father (during the last year and during childhood), but more to reactive than instrumental reasons to vicarious victimization by the father towards the mother (last year). Violence towards the mother responds to both reactive and instrumental reasons given that the difference in the scores for both types of reasons is not significant. In the case of girls, violence towards fathers and mothers responds to more reactive than instrumental reasons in all victimization experiences analyzed, with one exception. Specifically, violence towards mothers responds to both reactive and instrumental reasons for the direct victimization by the father in childhood.

## 4 Discussion

This study aimed to clarify the role of both lagged and simultaneous effects of exposure to violence at home (direct victimization and vicarious victimization) on CPV and the reactive or instrumental nature of these relation depending on the gender of both children and parents. To our knowledge, this is the first study to compare both time frames, which may help identify the explanatory mechanisms involved in this relationship.

The first objective was to analyze the relationships between CPV and lagged and simultaneous direct and vicarious family victimization. As expected (H1), positive and significant relationships were found between CPV and direct and vicarious victimization. Similar data are reported by other studies that have found a relationship between CPV and direct victimization and vicarious victimization (13, 18, 19, 21). This relationship is observed in victimization that occurred in the last year, as well as during childhood, and is present in both boys and girls. More specifically, the strongest correlations were found with direct victimization that occurred in the last year, suggesting that the immediate/simultaneous effects are more intense. These results are in line with other studies that have found a more strong relationship with direct victimization (8–10, 13). Furthermore, violence towards fathers correlates more stronger with victimization by father in boys and violence towards mothers correlates more stronger with victimization by mother in girls, suggesting a certain reciprocity between perpetrated violence and experienced violence, as well as a certain correspondence depending on the gender of the children and parents (10, 26).

The second objective was to identify the types of family victimization that best predict CPV. The obtained data show that, as expected (H2), both direct and vicarious victimization are significant predictors of CPV in boys and girls. This hypothesis is consistent with findings from other studies (9, 13, 14, 26). Furthermore, this is true whether victimization occurs close in time or distant in time. This explains both the distal and immediate effects of exposure to violence at home on CPV. Regarding which factor best predicts CPV, there are differences depending on the time frame, the type of victimization, and gender. In boys, the best predictor of violence towards the father and mother is direct victimization by the father during the last year. In the case of girls, the best predictor of violence towards the father is the victimization by the father during the last year and the best predictor of violence towards the mother is the victimization by the mother during the last year, indicating immediate or reciprocal effects. It also stands out that vicarious victimization is a predictor of CPV but mainly when it occurs during childhood, which seems to indicate more compensatory effects in this type of victimization. These findings point out three important aspects. On the one hand, although both time frame predicts CPV, more recent victimization exerts greater influence. Although some studies have confirmed the lagged effects (18) and simultaneous effects of exposure to violence (30), the data from the present study suggest that simultaneous victimization has greater explanatory weight. Secondly, direct victimization has more predictive weight than vicarious victimization, an aspect that has also been pointed out in other studies (9, 13). Finally, there are notable gender differences. While in boys the victimization by the father exerts more influence, in girls both parents exert influence, with correspondence between the person who experiences violence and the person who perpetrates it. Gender differences in the effect of vicarious victimization also draw attention. In the case of boys, the predictor variable of CPV is exposure to violence from the father towards the mother, while in the case of girls it is exposure to violence from the mother towards the father, primarily in both groups during childhood.

The third objective was to analyze what type of reasons for CPV (reactive, instrumental or both) intervene in the relationship between the different types of family victimization analyzed and CPV. The results show differences depending on gender. In boys, generally, CPV towards the father and mother is motivated by both reactive and instrumental reasons in all victimization experiences except one: the vicarious victimization of the father towards the mother during the last year in which reactive reasons are most prevalent. In the case of girls, violence towards fathers and mothers responds to more reactive than instrumental reasons in practically all the victimization experiences analyzed. Therefore, H3, referring to the fact that direct victimization would be related to CPV more through reactive than instrumental reasons (20), especially in the least year, is supported only in the case of girls. Additionally, there is correspondence according to the gender of the children and parents.

In short, the results provide empirical support for the two complementary models derived from social learning theory proposed to explain the relationship between child and parental violence. Parental violence towards children (direct victimization) occurring close in time appears to have the greatest explanatory weight in children’s violence towards parents, which supports the model of reciprocal effects or bidirectional violence. Future research should examine whether parent-child violence is really bidirectional by investigating at these dynamics in greater detail. Additionally, although to a lesser extent, exposure to violence between parents (vicarious victimization) during childhood is related to children’s violence towards parents, which supports the compensation model.

It is necessary to mention some limitations of the study that must be taken into account. Firstly, the participants in this research are Spanish adolescents, which affects the generalizability of the findings to other countries. Secondly, all measures were based on self-reports by adolescents, including the violence perpetrated by them and the violence they experienced and observed from their parents. To obtain a more complete view, it would be necessary to obtain information from parents or other sources of information. Lastly, it is necessary to take into account that it is a retrospective longitudinal study. Adolescents are asked to report events that occurred before the age of 10, which may introduce recall bias. Although it has been proven that the information provided about events that occurred years ago is valid (see review by Hardt and Rutter (41)), prospective longitudinal studies are necessary to support these relationships. Furthermore, it is necessary to clarify the direction of causal influences between the variables studied. The violence in both children and parents is not the same as one person’s violence in response to another’s violence. It is necessary to delve deeper into the complex relationships between the different types of violence, considering the family as an interconnected system, where the actions and relationships of each member impact the others. It would also be interesting to differentiate the types of violence (physical, psychological, economic and control/dominance) in future studies to find out if there is also correspondence between the types of violence suffered and exercised and if this occurs in both time frames.

Despite these limitations, the present study has provided relevant results on the effects of exposure to domestic violence on CPV. Firstly, the results indicate that both lagged and simultaneous effects of exposure to familiar violence are present in CPV, with the simultaneous effects being more intense. Secondly, both direct victimization and vicarious victimization predict CPV, with direct victimization having a stronger impact. Thirdly, the relationship between exposure to familiar violence and CPV can be explained by both reactive and instrumental reasons, with reactive reasons being more prominent, especially in girls.

These results have important implications for professional practice. Intervention in family contexts in which different forms of family violence are or have been present is essential to prevent the transmission of violence. Furthermore, in such contexts of exposure to violence, it is crucial to identify the underlying motivation for violence directed towards parents. The therapeutic approach varies depending on the reactive or instrumental nature of this type of violence. For reactive CPV, interventions should specifically focus on training cognitive and anger management strategies to generate alternative responses to aggression. For instrumental CPV, these interventions could include modifying dysfunctional beliefs about the use of violence and learning skills to resolve interpersonal conflicts.

## Data Availability

The raw data supporting the conclusions of this article will be made available by the authors, without undue reservation.
